# Novel Extraction Method Using Excipients to Enhance Yield of Genistein and Daidzein in *Trifolium pratensis* L.

**DOI:** 10.3390/pharmaceutics13060777

**Published:** 2021-05-22

**Authors:** Jurga Andreja Kazlauskaite, Liudas Ivanauskas, Jurga Bernatoniene

**Affiliations:** 1 Department of Drug Technology and Social Pharmacy, Lithuanian University of Health Sciences, LT-50161 Kaunas, Lithuania; jurga.andreja.kazlauskaite@stud.lsmu.lt; 2 Institute of Pharmaceutical Technologies, Lithuanian University of Health Sciences, LT-50161 Kaunas, Lithuania; 3 Department of Analytical and Toxicological Chemistry, Lithuanian University of Health Sciences, LT-50161 Kaunas, Lithuania; liudas.ivanauskas@lsmuni.lt

**Keywords:** *Trifolium pratensis* L., red clover, isoflavones, aglycones, excipients, extractions

## Abstract

Isoflavones can be found in different chemical forms, but the health beneficial effects mainly appear in their free forms—aglycones. Their yield in red clover (*Trifolium pratensis* L.) extracts differs due to different extraction and hydrolysis methodologies. The main aim of this study was to obtain the highest yields of daidzein and genistein from red clover blossoms through the various extraction and hydrolysis methods and to increase their quantities using additional excipients. Extracts were obtained by ultrasound-assisted, heat-reflux and maceration methods combining them with acidic, alkaline, and thermal hydrolysis. Using ultrasound-assisted extraction with optimal conditions and heat-reflux method highest yields of isoflavones were obtained in UTE510 (393.23 ± 19.66 µg/g daidzein and 171.57 ± 8.58 µg/g genistein); UTE530 (415.07 ± 20.75 µg/g daidzein and 150.57 ± 7.53 µg/g genistein) and HNE5 (432.30 ± 21.61 µg/g daidzein and 154.50 ± 7.72 µg/g genistein) samples. These conditions were used with excipients: magnesium aluminometasilicate, croscarmellose sodium, sodium carboxymethyl starch and vinylpyrrolidone-vinyl acetate copolymer. This is the first study reporting the ability of the vinylpyrrolidone-vinyl acetate copolymer to promote solubilization and availability of active compounds from a herbal extract, resulting in enhanced isoflavones yield. The results of the present study showing increased solubility and availability provided by the vinylpyrrolidone-vinyl acetate copolymer suggest that this preparation could in principle also reduce variability due to limited water solubility of isoflavones.

## 1. Introduction

Red clover (*Trifolium pratense* L.) is a perennial legume that is widely grown organically and conventionally in many countries because of its agriculture value or for their use in traditional medicine [1,2]. In the past decade, red clover has received a considerable amount of interest as an alternative valuable source of isoflavones with variety of health protective effects [3].

The main isoflavones found in the red clover are formononetin, biochanin A, daidzein and genistein [4]. These isoflavones possess estrogenic [5] and antiproliferative effects [6]. Various products from semi-purified isoflavones and their free forms have been studied for the possible preventive role of breast and prostate cancer, maintenance of bone health, improvement of cardiovascular health and benefits related to menopausal problems [5,7,8,9,10].

**Figure 1:**
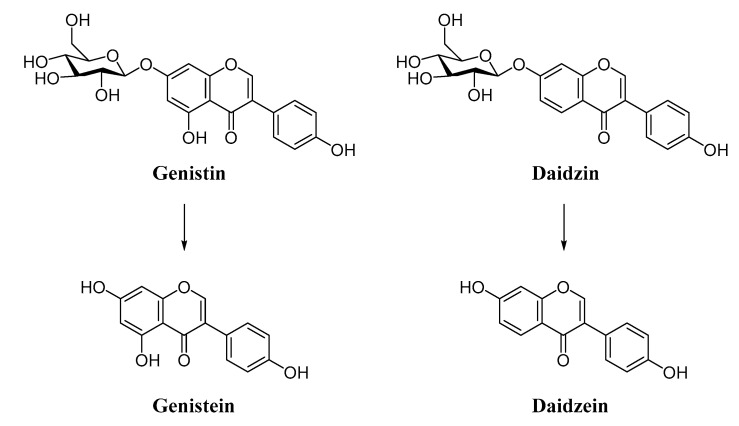
Chemical structures of genistin, genistein, daidzin and daidzein.

Natural isoflavone glycosides, such as daidzin and genistin are not easily absorbed in intestinal absorptive cells because of their large hydrophilic structures. These conjugated isoflavones are inactive compounds but become active in aglycone (daidzein and genistein) form when the glucose molecule is removed from the structure (Figure 1). The hydrolysis of glycosides is an important step to obtain biologically active and easily absorbed forms of isoflavones [11,12].

Choosing the right extraction method and parameters are the most important stages in the development of nutraceuticals from natural resources. Different extraction processes contribute to the extraction efficiency of the active ingredient from the solid matrix [13,14]. The extraction method of isoflavones daidzein and genistein from plant material should be simple, safe, inexpensive, and suitable for industrial applications. Isoflavonoids in plant material are mostly present as glycosides. Therefore, to extract the aglycone forms from plants, drastic methods (ultrasound/microwave-assisted extractions) or mild extraction techniques (maceration/percolation) followed by hydrolysis must be performed [15,16,17].

The conventional extraction methods, such as maceration, percolation and soxhlet extractions, have been employed for decades, but it is not economical because of relatively large quantities of solvent and the required long extraction times [18]. To reduce the time of extraction, many measures have been investigated, either separately or combined. Ultrasound-assisted extraction has been explored, changing various parameters, and it has shown a great increase of isoflavones yield, reduced quantities of solvents and shortened the time of extraction [15].

Additional transformation of isoflavone glycosides to aglycones can be achieved using an extraction method combined with hydrolysis. Using chemical (a base/an acid) or thermal (high temperatures) hydrolysis can increase aglycones content [19]. Using hydrolysis, glycosides convert to aglycones, but the temperatures and processing time should be closely monitored and properly selected because genistein and daidzein could degrade at high temperatures [20].

Excipients are the compounds that are added to the formulation along with pharmacologically active substances. The main purpose of adding them in drugs is to increase the bulk of the formulation along with imparting desired properties. Almost all drug dosage forms include an excipient to guarantee the dosage, stability and bioavailability [21,22]. Excipients can also be used for improving extraction and changing environmental conditions. For example, salts that change ion voltage, surfactants, emulsifiers (sorbitan esters (Spans^®^), polysorbates (Tweens^®^)), and pH-adjusting substances are used [23]. Croscarmellose sodium, sodium carboxymethyl starch and vinylpyrrolidone-vinyl acetate copolymer has been used for the first time as an excipient for chemical compound extractions; previously, it was used as an excipient in solid dosage forms [24,25,26]. Magnesium aluminometasilicate was used in previous studies to increase essential oil yield from nutmeg seeds [23].

So, the aim of this study was to establish an optimal extraction method, hydrolysis, and parameters to produce isoflavones from red clover and to use excipients on said extracts to increase the isoflavones yield.

## 2. Materials and Methods

### 2.1. Materials

Red clover samples were collected at Laičiai, Kupiškis district, northeast Lithuania. Flower buds and flowers (average color, light red, dark red and bright red) collections were made on 26 September. Samples were dried and stored at room temperature. Before use, clover flowers were grounded to a fine powder using Ultra Centrifugal Mill ZM 200 (Retsch, Haan, Germany). Grinding was performed at 4025 g using a 0.5 mm trapezoid holes sieve.

HPLC-grade and analytical-grade reagents were used: hydrochloric acid, sodium hydroxide, acetic acid, methanol, acetonitrile (Sigma Aldrich, Hamburg, Germany); standards of genistein, genistin, daidzein and daidzein (Sigma Aldrich, Steinheim, Germany); and ethanol (96%) (Vilniaus Degtine, Vilniaus, Lithuania). Purified water was prepared with GFL2004 (GFL, Burgwedelis, Germany). Deionized water was prepared with Milipore, SimPak 1 (Merck, Darmstadt, Germany). Excipients included croscarmellose sodium, sodium carboxymethyl starch and vinylpyrrolidone-vinyl acetate copolymer (JRSPharma & Gujarat Microwax Pvt Ltd., Ahmedabad, India) and magnesium aluminometasilicate (Neusilin^®^) (US2, Fuji Chemical Industries Co., Ltd., Toyoma, Japan).

### 2.2. Extraction of Plant Material

#### 2.2.1. Moisture Determination of Red Clover Plant Matearial

The moisture content of the milled red clover flowers was determined using a KERN MLB apparatus (KERN & Sohn GmbH, Balingen, Germany). A total of 0.3 ± 0.01 g grams of the material was placed in the apparatus and heated to 105 °C. At the end of the operation, the device provided a calculated moisture content of the material [27]. The moisture of the red clover plant material humidity ranged from 7% to 7.4%.

#### 2.2.2. Maceration Extraction (ME)

**Figure 2:**
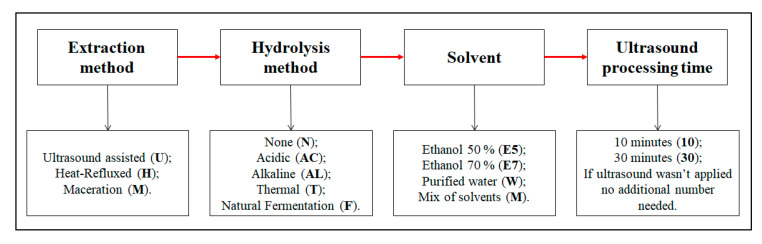
Decoding of the samples provided in Table 1.

Maceration extraction was carried out using a modified method of Krähmer et al., 2013 [28]. A total of 0.3 ± 0.001 g of dried and milled flower heads were macerated in 10 mL ethanol (70 or 50% *v*/*v*). The samples were centrifuged for 10 min at 3382 g, followed by the decantation of the supernatant. The extracts were hydrolyzed using alkaline hydrolysis and then filtered through PVDF syringe filters (pore size 0.22 μm) for further HPLC analysis. The extraction conditions are displayed in Table 1 and decoding of the samples are provided in Figure 2.

#### 2.2.3. Ultrasound-Assisted Extraction (UAE)

Ultrasound-assisted extraction was performed using an ultrasound bath (frequency 38 kHz) (Cambridge, UK, Grant Instruments™ XUB12 Digital). A total of 0.3 ± 0.001 g of dried and milled flower heads was macerated in 10 mL of solvent. The extraction of isoflavones was performed by employing different extraction conditions—solvent (70 or 50% ethanol and purified water *v*/*v*) and extraction time: 10 to 30 min, processing temperature 40 ± 2 °C [17,29]. The samples were centrifuged for 10 min at 3382 g, followed by the decantation of the supernatant. The extracts were hydrolyzed and then filtered through PVDF syringe filters (pore size 0.22 μm) for further HPLC analysis. The extraction conditions are displayed in Table 1 and decoding of the samples are provided in Figure 2.

For easier comprehension, the samples are coded according to their conditions (Figure 2). The first letter of the sample indicates extraction method; the second, the hydrolysis method; and the third, the solvent and ultrasound processing time (if ultrasound was not applied, no additional number was added).

**Figure 3:**
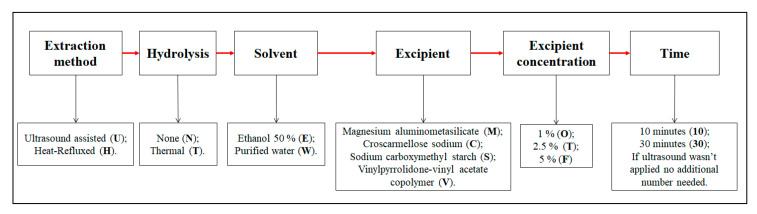
Decoding of the samples provided in Table 2.

Some of the samples were modified and prepared with vinylpyrrolidone-vinyl acetate copolymer, croscarmellose sodium, sodium carboxymethyl starch or magnesium aluminometasilicate. The extracts were made in the same conditions, which were listed earlier. Sample preparation conditions are listed in Table 2 and decoding of the samples are provided in Figure 3.

The samples in Table 2 are coded according to their conditions (Figure 3). The first letter of the sample indicates the extraction method; the second, the hydrolysis method; the third, the solvent; the fourth, the excipient; and the fifth, the excipient concentration and the number show ultrasound processing time (if ultrasound was not applied, no additional number was added).

Purified water or 50% of ethanol (*v*/*v*) was used as the solvent and the excipient was added to the extraction mixture. The excipients concentration in the extract were 1% (*v*/*w*) (0.1 ± 0.001 g was added to the extraction mixture of 10 mL); for vinylpyrrolidone-vinyl acetate copolymer, it was from 1 to 5% (*v*/*w*) (0.1 ± 0.001–0.5 ± 0.001 g were added to the extraction mixture of 10 mL). The excipient amount was based on solvent quantity. The samples were centrifuged for 10 min at 3382 g, followed by the decantation of the supernatant. The extracts were filtered through PVDF syringe filters (pore size 0.22 μm) prior to HPLC analysis.

#### 2.2.4. Heat-Reflux Extraction (HRE)

A total of 0.3 ± 0.001 g of dried and milled flower heads were mixed with 10 mL of used solvent (70%, 50% ethanol or purified water *v*/*v*) in a 250 mL round bottom flask and it was refluxed in the sand bath at 100 °C for 1 h. Consequently, the mixture was left to cool at 25 ± 2 °C temperature. The samples were centrifuged for 10 min at 3382 g, followed by decantation of the supernatant. The extracts were filtered through PVDF syringe filters (pore size 0.22 μm) prior to HPLC analysis. The extraction conditions are displayed in Table 1.

### 2.3. Hydrolysis and Neutralization

#### 2.3.1. Acidic Hydrolysis and Neutralization

For the acid hydrolysis modified method of Zgórka, 2009 was used [4]. Extracts were transferred to a 250 mL round-bottom flask. A total of 37% HCl was added to the whole medium ratio 1:12 (*v*/*v*) and finally the flask was placed in a heating mantle under a reflux condenser. Sample hydrolysis was performed from the beginning of liquid boiling for a period of 1 h. Then, hydrolyzed extracts were cooled down to 25 ± 2 °C then neutralized to pH∼2.5 by adding aqueous solution of 2 M NaOH while stirring. The neutralized extracts were filtered. The neutralized solution was prepared for further HPLC analysis.

#### 2.3.2. Alkaline Hydrolysis and Neutralization

Alkali hydrolysis was carried out using 25% NaOH. The pH was changed to 10.5 and then the extracts were sonicated at 45 ± 2 °C for 10 min. After hydrolysis, the sample was neutralized to pH 5.7 using 25% acetic acid. The neutralized extracts were filtered. The neutralized solution was prepared for further HPLC analysis.

#### 2.3.3. Thermal Hydrolysis

Thermal hydrolysis was carried out by transferring the extract to a 250 mL round-bottom flask. It was refluxed in the sand bath at 100 °C for 1 h. After that, the mixture was left to cool at 25 ± 2 °C temperature. The samples were centrifuged for 10 min at 3382 g, followed by the decantation of the supernatant. The extracts were filtered through PVDF syringe filters (pore size 0.22 μm) prior to HPLC analysis.

#### 2.3.4. Maceration Extraction (ME) with Natural Hydrolysis

The maceration was carried out using 0.3 ± 0.001 g of dried and milled flower heads, which were weighed and covered completely with 30 mL deionized water and kept overnight. The next day, extracts were filtered using a Buchner funnel and filtrates were collected. A second overnight water extraction was carried out using 20 mL of deionized water. The spent plant material was extracted again overnight with 40 mL of 96% ethanol and the fourth time with 70% aqueous ethanol. All four filtrates from each sample were combined as one extract [1]. The extraction conditions are displayed in Table 1.

### 2.4. HPLC–PDA Conditions

HPLC analyses have been carried out using the Shimadzu Nexera X2 LC-30AD HPLC system (Shimadzu, Tokyo, Japan), consisting of a quaternary pump, an on-line de-gasser, a column temperature controller, the SIL-30AC autosampler (Shimadzu, Tokyo, Japan) equipped with the CTO-20AC thermostat (Shimadzu, Tokyo, Japan) as well as the SPD-M20A diode array detector (DAD). For determination of polyphenols, an ACE 5 C18 250 × 4.6 mm column (Advanced Chromatography Technologies, Aberdeen, Scotland) was used. The mobile phase consisted of solvent A (acetic acid/methanol/deionized water) (1:10:89 *v*/*v*/*v*) and solvent B (acetic acid/methanol) (1:99 *v*/*v*/*v*). The linear gradient elution profile was as follows: 80% A/20% B at 0 min, 30% A/70% B at 30 min, 90% A/10% B at 39 to 40 min. The flow rate was 1 mL/min, and the injection volume was 10 μL. Absorption was measured at 260 nm. Quantification of isoflavone compounds was performed using reference standards of daidzein, genistein, daidzin, and genistin. The range of linearity of daidzein was 0.43 to 221 µg/mL, genistein was 0.43 to 218 µg/mL, daidzin was 0.32 to 165 µg/mL, and genistin was 0.3 to 151.5 µg/mL. The linearities of the calibration curves are provided in Table 3. The contents were expressed as μg/g dry weight (dw). Specificity is the ability to unequivocally assess the analyte in the presence of components, which may be expected to be present. In this study, standards (genistein, daidzein, genistin, daidzin) were analyzed and their retention time and spectra were compared with prepared extracts [27].

### 2.5. Statistical Analysis

Data is presented as the mean ± standard deviation (SD). All experiments were performed in triplicate. Statistical analysis of the results was performed with SPSS 20.0 (IBM Corporation, Armonk, NY, USA). One-way ANOVA was used to investigate the differences between extractions. Post hoc comparisons of the means were performed according to Tukey’s HSD test. The means of compared samples were considered significantly different when *p* < 0.05.

## 3. Results and Discussion

### 3.1. Determination of Isoflavones Aglycones in Trifolium pratensis L. Extracts

#### 3.1.1. Aglycones Extraction Using UAE Method

**Figure 4:**
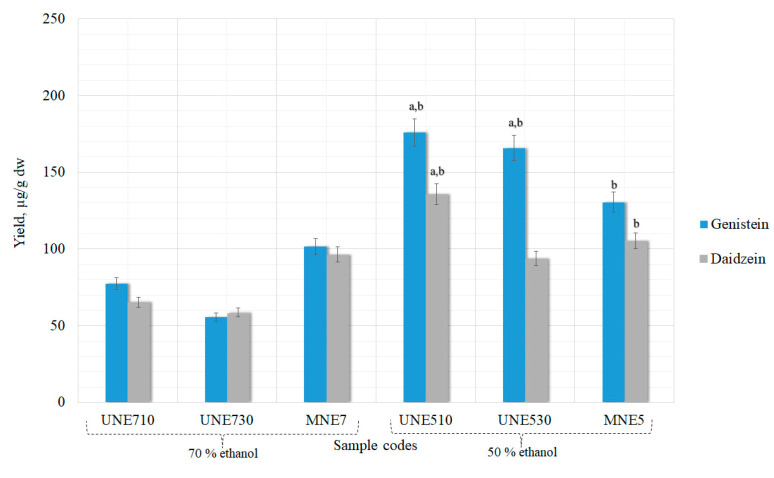
Influence of UAE treatment time and solvent concentration without hydrolysis on the maximal extraction yield of genistein and daidzein in extracts. ^a^ *p* < 0.05 vs maceration with 50% ethanol (MNE5); ^b^ *p* < 0.05 vs maceration with 70% ethanol (MNE7). Sample codes and preparation conditions are displayed in Table 1.

The yield of daidzein and genistein were determined in the different concentrations ethanolic extracts obtained from dried *Trifolium pratensis* L. flower heads material by UAE. Isoflavones were determined using HPLC-PDA. Conventional extraction methods are based on the use of chemical solvents and sample heating to maximize the solubility of the active principles and speed up mass transfer. The extraction yield depends on several factors, including the type, concentration and amount of solvent, its residence time and temperature [30]. Different processing times (10 and 30 min) and two ethanol concentrations (50% and 70%) were employed for flower heads extraction using UAE (temperature 40 °C). These extraction conditions were used to determine the effect of treatment time and solvent concentration on isoflavones content when the hydrolysis is not involved. In the study by L. Y. Yoshiara et al., it was determined that using pure organic solvents for isoflavone extraction was not efficient, suggesting that the use of these extraction solvents in binary or ternary mixtures with water could be more convenient [31]. Additionally, Rostagno et al.’s study concluded that the best solvent for ultrasound-assisted extraction of isoflavones is 50% ethanol [32]. Therefore, based on conducted and published studies, it was decided to use two ethanol concentrations—50% and 70%—with water (*v*/*v*) as a safe solvent, so that the extracted isoflavones could later be used in nutraceuticals production. The results of genistein and daidzein yields, using only UAE method without hydrolysis, are shown in Figure 4.

The highest genistein and daidzein amounts were obtained using 50% ethanol and 10 min ultrasound processing time −175.93 ± 8.7 and 135.60 ± 6.7 µg/g (genistein and daidzein, respectively). Extending the processing time from 10 to 30 min aglycones quantities decreased in all the samples. The driving force for UAE is cavitation. Decreasing yields of compounds when the extraction time is increased can be explained by cavitation bubbles collapse [33]. As soon as a bubble collapses near a surface (cell walls, herbal particles, or any suspended material in the liquid), it deforms, taking up a doughnut shape, impacting the wall with the potential to sweep particles away from the surface or indeed cause actual damage. During ultrasound extraction, the solvent vapors and any gases dissolved in the solvent that are in the bubble are exposed to the extreme conditions generated by collapse. If there is water vapor in the bubble, its collapse leads to the homolytic splitting of the water molecules to generate reactive HO· and hydrogen atoms. The radicals formed then undergo reactions to produce H_2_O_2_ and other active oxidizing agents [34]. The amount of generated oxidizing agents during processing is small, but it could cause some degradation of the extract if the sonication continued over a long period [35].

The differences between simple maceration (MNE5) and UAE samples when 50% concentration ethanol was used were statistically significant in the sample UNE510 and in the sample UNE530 only genistein yield was significant (Figure 4). The same results were precured when comparing UNE510 and UNE530 samples with maceration (MNE7) that was carried out using 70% concentration ethanol.

Low amounts of the glycosides genistin and daidzin were obtained in the samples; their yields were statistically insignificant (*p* > 0.05). Therefore, they are not shown in the graph.

#### 3.1.2. Aglycones Extraction Using UAE Method with Acidic Hydrolysis

**Figure 5:**
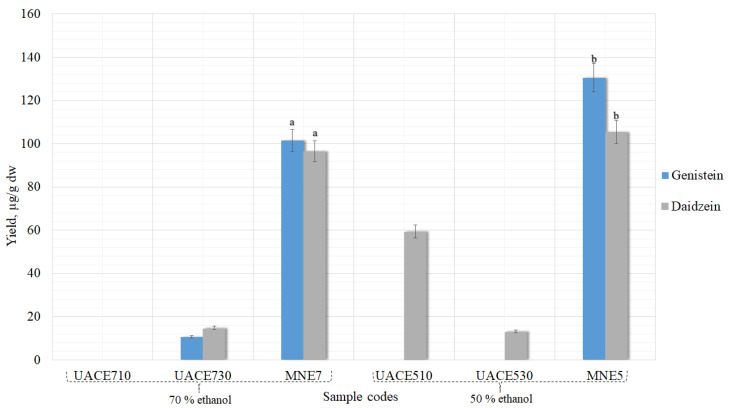
Influence of UAE treatment time and solvent concentration with acidic hydrolysis on the maximal extraction yield of genistein and daidzein in extracts. ^a^ *p* < 0.05 vs. maceration with 50% ethanol (MNE5); ^b^ *p* < 0.05 vs. maceration with 70% ethanol (MNE7). Sample codes and preparation conditions are displayed in Table 1.

Combining UAE with acidic hydrolysis and using 70% or 50% concentration ethanol as a solvent, genistein was not found (Figure 5). The exception was sample UACE730, but the amount of genistein was low (10.67 ± 0.53 µg/g) and, compared to maceration, statistically insignificant (*p* > 0.05).

During acidic hydrolysis, heating is required. Using hydrochloric acid, the samples were hydrolyzed from glycosides to aglycones; however, in high temperatures, genistein degrades [36]. Therefore, the heating in acidic conditions was too long, because no genistin or genistein was found in the samples (Table 4). Chemical structure of isoflavones dictates their stability under variable pH and temperature conditions. Genistein loss could be due to either a complete degradation or a transformation into isoflavone derivative [37]. Relatively large amounts of daidzin, compared to MNE5 and MNE7 samples, were found in the samples UACE710, UACE730, UACE510 and UACE530 (Table 4), indicating that the hydrolysis of these samples was not fully complete. Although no genistein remained during hydrolysis, it was not sufficient for the complete conversion of daidzin to daidzein; consequently, this method was not sufficient to obtain both aglycones.

In the research by Gikas et al., 2008, red clover extraction was proceeded using HCl, but they did not perform neutralization [38]. Daidzein levels were similar, but genistein levels were different. The extracted amount of genistein was not very high (0.11 mg/g), but it did not degrade as in this experiment. The difference between these two experiments were UAE use. Ultrasound was not used in the study described in the article, so it can be speculated that a combination of ultrasound and heating in an acidic medium were too harsh for genistein extraction. The paper also suggested that later-harvested clover has higher levels of daidzein and lower levels of genistein. This trend was also observed in this study.

#### 3.1.3. Aglycones Extraction Using UAE Method with Alkaline Hydrolysis

**Figure 6:**
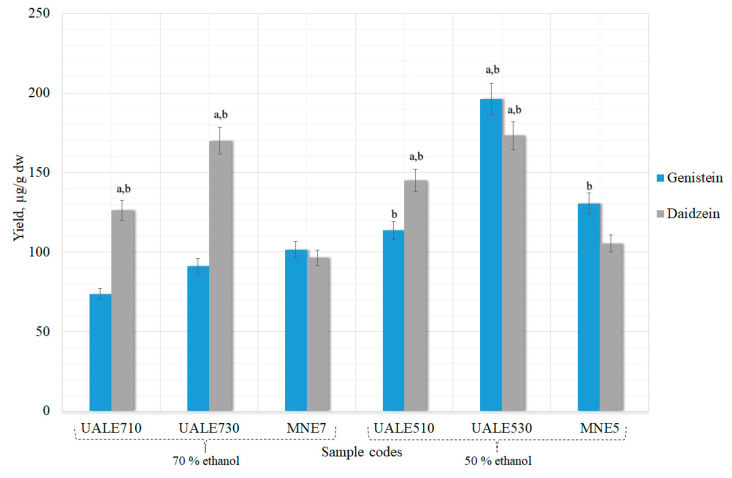
Influence of UAE treatment time and solvent concentration with alkaline hydrolysis on the maximal extraction yield of genistein and daidzein in extracts. ^a^ *p* < 0.05 vs. maceration with 50% ethanol (MNE5); ^b^ *p* < 0.05 vs. maceration with 70% ethanol (MNE7). Sample codes and preparation conditions are displayed in Table 1.

During alkaline hydrolysis extending sonication time genistein yields increased (Figure 4). This tendency was also observed with daidzein when the solvent was 50% ethanol. Higher amounts of aglycones were obtained during alkaline than acid hydrolysis. Alkaline hydrolysis also yielded higher amounts of isoflavones than ultrasound alone. The sample that contained the most isoflavones was UALE530 (196.30 ± 9.8 and 173.10 ± 8.6 µg/g genistein and daidzein, respectively); it was significantly higher (*p* < 0.05) than when extracted using maceration (Figure 6). Lower but similar results were found in the UALE510 sample (*p* < 0.05).

Evaluating the results obtained by the UAE method with or without chemical hydrolysis, it was found that statistically significant amounts of aglycones are present in the extracts when 50% concentration ethanol solvent is used. Therefore, only this ethanol concentration was used in further samples.

#### 3.1.4. Aglycones Extraction Using UAE, HRE, ME and ME with Natural Fermentation

**Figure 7:**
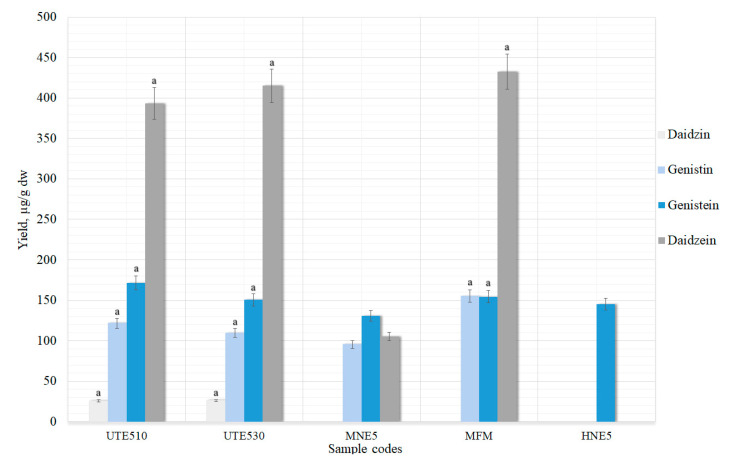
UAE treatment with thermal hydrolysis, HRE, ME and ME with natural hydrolysis on the maximal extraction yield of genistein and daidzein in extracts. ^a^ *p* < 0.05 vs. maceration with 50% ethanol (MNE5). Sample codes and preparation conditions are displayed in Table 1.

In sample MFM (Figure 7), extracted using ME with natural fermentation, no daidzein was detected, but a similar amount of genistein was found compared to other methods. This method was performed at 25 ± 2 °C, but the enzyme *β*-glucosidase that can be found in the red clover grounded powder converts glucosides to aglycones and possesses the highest activity at 45 °C. *β*-glucosidase is stable in high temperatures and does not denature for a long time [39]. During natural hydrolysis, the temperature was not high enough for the enzymes to remove glucoside groups. In the literature, it was observed that not only can enzymes break the glycosides down, but it can also be achieved using heat and the reaction proceeds faster [40].

Thermal hydrolysis with the UAE method gave the best results compared with the UAE method without hydrolysis, with acidic or alkaline hydrolysis (Figure 7). Samples UTE510 and UTE530 yielded statistically significant results (*p* < 0.05) compared to the maceration (MNE5) sample. Glycosides daidzin and genistin were also present in these samples (Figure 7). Increasing the sonication time daidzein (from 393.23 ± 19.66 (UTE510) to 415.07 ± 20.75 (UTE530) µg/g) and daidzin (26.00 ± 1.30 (UTE510) to 28.1 ± 1.41 (UTE530) µg/g) amounts increased, but genistein (from 171.57 ± 8.57 (5.4) to 150.57 ± 7.52 (6.4) µg/g) and genistin (from 121.60 ± 6.08 (UTE510) to 109.70 ± 5.49 (UTE530) µg/g) decreased (Figure 7). The HRE method also yielded the highest results (*p* < 0.05) for daidzein in sample HNE5 (432.30 ± 21.6 µg/g) compared to all previous extraction methods and genistein did not degrade during heating (154.5 ± 7.7 µg/g).

In the research by Booth, Overk, Yao, Totura, et al., 2006, genistein and daidzein yields from red clover flower heads or aboveground parts extracts vary widely, but in very small amounts [1]. Comparing the yields of these two studies, the ranges of daidzein and genistein amounts obtained in this study are 10 times higher than the reported results. It was also observed that the amounts of isoflavones depends not only on the method of extraction, the growth stage of the plant or the part of the plant, but also on the growth conditions (growth temperature, humidity, soil fertility). As a result, the yield ranges of isoflavones can be very wide [3].

### 3.2. Selection of the Excipients

**Figure 8:**
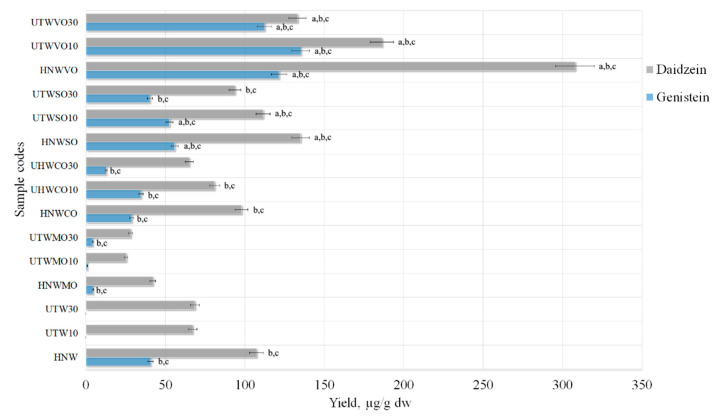
Quantitative yield of isoflavone aglycones using excipients (1%). Control samples without excipients (HNW; UTW10; UTW30), samples with magnesium aluminometasilicate (HNEMO; UTEMO10; UTEMO30), croscarmellose sodium (HNWCO; UHWCO10; UHWCO30), sodium carboxymethyl starch (HNWSO; UTWSO10; UTWSO30) and vinylpyrrolidone-vinyl acetate copolymer (HNWVO; UTWVO10; UTWVO30). ^a^ *p* < 0.05 vs. HNW, ^b^ *p* < 0.05 vs. UTW10, ^c^ *p* < 0.05 vs. UTW30. Sample codes and preparation conditions are displayed in Table 2.

Excipients may improve the solubility of certain active substances in poorly water-soluble drugs [41]. Therefore, it was decided to use excipients during the extractions and to determine whether they could increase the yields of isoflavones. Most isoflavones were obtained using ultrasound (40 °C, 10 to 30 min, 50% ethanol) in combination with thermal hydrolysis or using HRE alone. Therefore, these extraction conditions will be applied using additional compounds to improve the solubility of isoflavones. Samples prepared using UAE for 10 min (i.e., UTW10) or 30 min (i.e., UTW30) were combined with thermal hydrolysis or only HRE (i.e., HNW) alone. Experiment was carried out using excipients (1%) and purified water as a solvent (Figure 8).

Excipients can be natural, synthetic or semisynthetic compounds that play a vital part in pharmacological products [41]. Magnesium aluminometasilicate, croscarmellose sodium, sodium carboxymethyl starch and vinylpyrrolidone-vinyl acetate copolymer as excipients can improve the oral bioavailability of poorly water-soluble drugs by enhancing the solubility and drug release [42]. The use of modern carriers with a large specific surface area and high absorption capacity is a good way of incorporating higher doses of water-insoluble or poorly soluble compounds into liquid–solid systems and increase their bioavailability. The selected different excipients were expected to absorb isoflavones during extraction and increase their final yields.

When purified water was used as a solvent with the excipient croscarmellose sodium, sodium carboxymethyl starch or vinylpyrrolidone-vinyl acetate copolymer, the yield of isoflavones significantly increased compared to the control samples (Figure 8). Glycosides, daidzin and genistin were not obtained with the use of excipients. Excipient magnesium aluminometasilicate reduced the amount of isoflavones, compared to controls, prepared using the same conditions (Figure 8). In the literature, magnesium aluminometasilicate increased essential oil yield and quantities of various compounds in it [23]. Therefore, possibly, magnesium aluminometasilicate absorbed terpenes, but not isoflavones from red clover extracts.

Using the excipients, statistically significant yields of aglycones were obtained with 1% vinylpyrrolidone-vinyl acetate copolymer. Using the HRE method, aglycones yields found in HNWVO sample were 307.80 ± 15.39 µg/g daidzein and 121.40 ± 6.07 µg/g genistein (Figure 8). Isoflavones yields decreased using UAE with thermal hydrolysis, and this correlation was observed in all the samples with excipients.

As the results show (Figure 8), lower amounts of isoflavones were obtained with croscarmellose sodium and sodium carboxymethyl starch compared to vinylpyrrolidone-vinyl acetate copolymer. Sodium carboxymethyl starch samples UTWSO10 (52.50 ± 2.63 µg/g genistein and 111.40 ± 5.57 µg/g daidzein) and UTWSO30 (40.23 ± 2.01 µg/g genistein and 93.57 ± 4.68 µg/g daidzein) showed statistically significant results compared to the control samples, but the yields of aglycones was not as high as using vinylpyrrolidone-vinyl acetate copolymer. Therefore, the use of excipients has great scope for improving the methodology of isoflavones extraction.

### 3.3. Vinylpyrrolidone-Vinyl Acetate Copolymer Determination of the Optimal Concentration for Higher Amounts of Aglycones Using Purified Water

When purified water is used as a solvent with an excipient vinylpyrrolidone-vinyl acetate copolymer, the same amounts of isoflavones can be obtained as with 50% ethanol under the same conditions. To test whether the yields of isoflavones obtained in water could be increased, the amounts of excipient added to the extract were increased. It was decided to use 1, 2.5 and 5% (*v*/*w*) vinylpyrrolidone-vinyl acetate copolymer for extractions.

**Figure 9:**
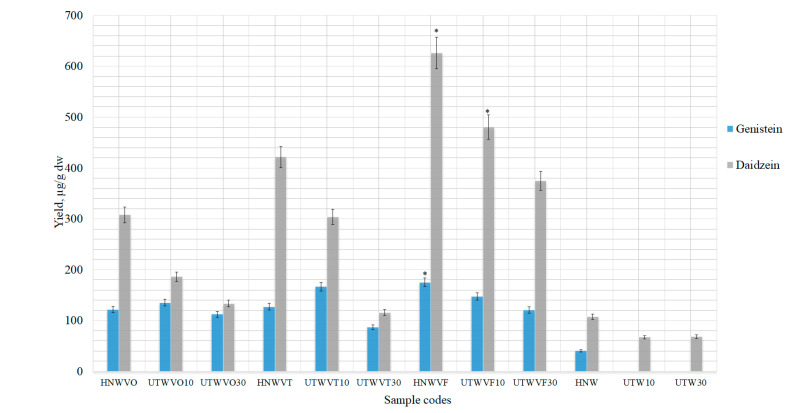
Quantitative yield of isoflavone aglycones using different amounts (1, 2.5 and 5%) of vinylpyrrolidone-vinyl acetate copolymer for extractions. Control samples without excipients (HNW; UTW10; UTW30), 1% of excipient (HNWVO; UTWVO10; UTWVO30), 2.5% (HNWVT; UTWVT10; UTWVT30) and 5% (HNWVF; UTWVF10; UTWVF30). * *p* < 0.05 vs. HNE5 sample prepared HRE method. Sample codes and preparation conditions are displayed in Table 2.

After extractions with different amounts of vinylpyrrolidone-vinyl acetate copolymer, all data obtained were statistically significant compared to controls (HNW; UTW10; UTW30) (Figure 9). For this reason, the data was compared with the sample that yielded the highest amounts of isoflavones in this study, which was a HNE5 sample prepared using the HRE method.

The best results were obtained with a sample using 5% excipient (Figure 9). The yields of isoflavones obtained were higher than the best result obtained in the whole study, which was previously determined in sample HNE5 (432.30 ± 21.6 µg/g and 154.5 ± 7.7 µg/g daidzein and genistein, respectively) (Figure 7). However, HNE5 sample was prepared using 50% ethanol, not purified water. The results were statistically significant when comparing with HNE5 sample in the HNWVF (626.10 ± 31.35 µg/g and 175.56 ± 8.7 µg/g daidzein and genistein, respectively) (Figure 9). In the UTWVF10 sample, only the amount of daidzein was statistically significant (480.36 ± 24.01 µg/g and 147.23 ± 7.36 µg/g daidzein and genistein, respectively). Increasing the amounts of excipient, the solubility of the resulting isoflavones in the aqueous solvent increases. This could create an opportunity to use cheaper, safer solvents (such as purified water), but obtain the same amounts of isoflavones as using expensive solvents without excipients. Therefore, it would still be possible to try to increase the excipient concentrations in water and to set a maximum concentration at which maximum levels of aglycones could be obtained; this could be a future goal for further research.

### 3.4. Vinylpyrrolidone-Vinyl Acetate Copolymer Use with Ethanol

**Figure 10:**
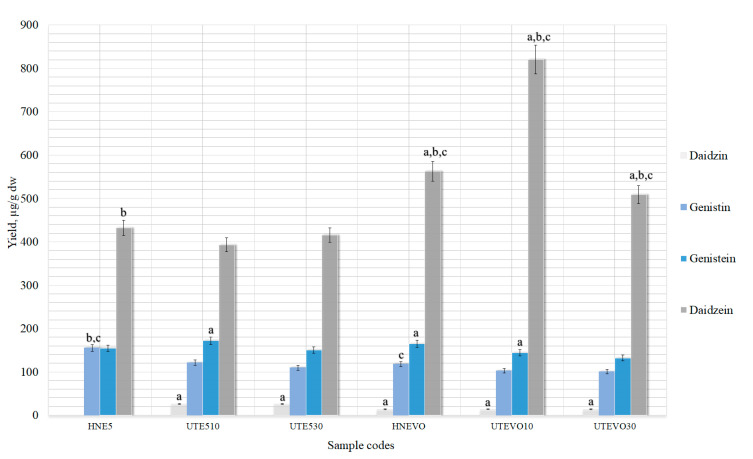
Quantitative yield of isoflavone aglycones using 1 of vinylpyrrolidone-vinyl acetate copolymer for extractions in 50% ethanol. Control samples without excipients (HNE5; UTE510; UTE530), 1% of excipient (HNEVO; UTEVO10; UTEVO30), ^a^ *p* < 0.05 vs. HNE5 sample; ^b^ *p* < 0.05 vs. UTE510, ^c^ *p* < 0.05 vs. UTE530. Sample codes and preparation conditions are displayed in Table 2.

High concentrations of isoflavone aglycones were obtained using 5% vinylpyrrolidone-vinyl acetate copolymer in water extracts. Comparing the control samples PDV1-3 produced in water under the same conditions as HNE5, UTE510 and UTE530 using 50% ethanol, it was found that the samples obtained in water had statistically lower values (Figure 10). Therefore, due to the use of excipients with water, we decided to determine the amounts of isoflavones under the same conditions as 50% ethanol and 1% vinylpyrrolidone-vinyl acetate copolymer.

As shown in Figure 10, no isoflavones glycosides were found in the aqueous extracts. Though, using 50% ethanol as a solvent in the extracts, both genistin and daidzin were detected (Figure 10). Daidzein yields in all three samples with vinylpyrrolidone-vinyl acetate copolymer (HNEVO; UTEVO10; UTEVO30) in ethanol were statistically significant when comparing them with controls. UTEVO10 contained the highest daidzein yield in this study—820.50 ± 41.02 µg/g. Genistein yields were lower, but the HNEVO and UTEVO30 sample results were statistically significant compared to the control sample HNE5 (Figure 10). Samples prepared with excipient and ethanol had higher levels of isoflavones, both aglycones and glycosides. As mentioned before, samples prepared in water with the excipient showed similar yields of isoflavones as the samples prepared under the same conditions with ethanol, but without excipients. Although 50% ethanol increased the amount of daidzein compared to the 5% excipient used in water (Figure 9), there was no significant increase in genistein levels. Therefore, the use of excipient vinylpyrrolidone-vinyl acetate copolymer in ethanol mainly yielded glycosides (Figure 10) and aglycone daidzein from plant material.

## 4. Conclusions

After applying different extraction methods and hydrolysis, it was determined that, using UAE with optimal conditions (processing 10 or 30 min combined with thermal hydrolysis) and the HRE method, the highest extractions of isoflavones were obtained in samples UTE510 (393.23 ± 19.66 µg/g daidzein and 171.57 ± 8.58 µg/g genistein), UTE530 (415.07 ± 20.75 µg/g daidzein and 150.57 ± 7.53 µg/g genistein) and HNE5 (432.30 ± 21.61 µg/g daidzein and 154.50 ± 7.72 µg/g genistein). These conditions were used with excipients.

In this work, the use of different excipients during the extractions was performed as an effective strategy to enhance isoflavones yield in red clover extracts. This is the first study reporting the ability of the vinylpyrrolidone-vinyl acetate copolymer to promote solubilization and availability of active compounds from a herbal extract, resulting in enhanced isoflavones yield. Using 1% vinylpyrrolidone-vinyl acetate copolymer (HNWVO sample, 307.80 ± 15.39 µg/g and 121.40 ± 6.07 µg/g daidzein and genistein, respectively) in the production of copolymer extracts using water as solvent, it was determined that the amounts of genistein obtained were similar as using 50% ethanol as solvent. Increasing the amount of this excipient to 5%, isoflavone yield further increased (HNWVF sample) to 626.10 ± 31.35 µg/g daidzein and 175.56 ± 8.7 µg/g genistein. The results of the present study showing increased solubility and availability provided by the vinylpyrrolidone-vinyl acetate copolymer suggest that this preparation could in principle also reduce variability due to limited water solubility of isoflavones. By changing the solvent to 50% ethanol, the highest statistically significant yields of isoflavones in this study were obtained in the sample UTEVO10 820.50 ± 41.02 and 144 ± 7.22 µg/g daidzein and genistein, respectively.

The resulting isoflavone-rich extracts could be used in the production of various pharmaceutical forms, as recent studies suggest, with a possible preventive role in breast and prostate cancer, improvement of cardiovascular health or benefits related to menopausal problems.

## Figures and Tables

**Table 1:** Extraction conditions used for the experiment.

Sample Code	Method *	Hydrolysis Method	Solvent	Processing/Extraction Time, min
UNE710	U	-	ethanol 70%	10
UACE710		acidic		10
UALE710		alkaline		10
UNE730		-		30
UACE730		acidic		30
UALE730		alkaline		30
UNE510	U	-	ethanol 50%	10
UACE510		acidic		10
UALE510		alkaline		10
UTE510		thermal		10
UNE530		-		30
UACE530		acidic		30
UALE530		alkaline		30
UTE530		thermal		30
HNW	H	-	purified water	60
UTW10	U	thermal	purified water	10
UTW30				30
MNE5	M	-	ethanol 50%	1080
MNE7		-	ethanol 70%	
MFM	M	natural fermentation	70%; 96% ethanol, deionized water	2880
HNE5	H	-	ethanol 50%	60

* U—Ultrasound-assisted extraction; H—Heat-reflux extraction; M—maceration.

**Table 2:** Extraction conditions using excipients for the experiment.

Sample Code	Extraction Method *	Hydrolysis Method	Solvent	Excipient	Excipient: Extract Ratio	Processing Time, min
UTEMO10	U	thermal	ethanol 50%	Magnesium aluminometasilicate	1:100	10
UTEMO30						30
HNEMO	H	-				60
UTWMO10	U	thermal	purified water		1:100	10
UTWMO30						30
HNWMO	H	-				60
UHWCO10	U	thermal	purified water	Croscarmellose sodium	1:100	10
UHWCO30						30
HNWCO	H	-				60
UTWSO10	U	thermal	purified water	Sodium carboxymethyl starch	1:100	10
UTWSO30						30
HNWSO	H	-				60
UTWVO10	U	thermal	purified water	Vinylpyrrolidone-vinyl acetate copolymer	1:100	10
UTWVO30						30
HNWVO	H	-				60
UTWVT10	U	thermal	purified water		2.5:100	10
UTWVT30						30
HNWVT	H	-				60
UTWVF10	U	thermal	purified water		5:100	10
UTWVF30						30
HNWVF	H	-				60
UTEVO10	U	thermal	ethanol 50%		1:100	10
UTEVO30						30
HNEVO	H	-				60

* U—ultrasound-assisted extraction; H—heat-reflux extraction; M—maceration.

**Table 3:** The linearities of calibration curves of isoflavones.

Component	Calibration Equation	Coefficient of Determination *R*^2^	Coefficient of Correlation *R*	LOD *	LOQ **
Daidzein	59,664.2× + 37,164.6	0.9999	0.9999	0.05	0.12
Genistein	73,083.1× + 44,202.9	0.9999	0.9999	0.05	0.12
Daidzin	38,202.1× + 19,377.4	0.9999	0.9999	0.08	0.31
Genistin	49,602.9× + 24,083.3	0.9999	0.9999	0.075	0.28

* LOD—limit of detection; ** LOQ—limit of quantification.

**Table 4:** Isoflavone glycosides genistin and daidzin yields (µg/g) found in samples treated by acid hydrolysis.

Sample Code	Genistin, µg/g dw	Daidzin, µg/g dw
UACE710	0.00 ± 0.00	221.37 ± 11.06
UACE730	0.00 ± 0.00	94.37 ± 4.71
MNE7	95.67 ± 4.78	0.00 ± 0.00
UACE510	0.00 ± 0.00	15.33 ± 0.76
UACE530	0.00 ± 0.00	43.80 ± 2.34
MNE5	95.40 ± 4.77	0.00 ± 0.00

## Data Availability

The data presented in this study are available on request from the corresponding author.

## References

[1] Booth N.L., Overk C.R., Yao P., Totura S., Deng Y., Hedayat A.S., Bolton J.L., Pauli G.F., Farnsworth N.R. (2006). Seasonal Variation of Red Clover (Trifolium Pratense L., Fabaceae) Isoflavones and Estrogenic Activity. J. Agric. Food Chem..

[2] Sabudak T., Guler N. (2009). *Trifolium* L.-A Review on Its Phytochemical and Pharmacological Profile. Phyther. Res..

[3] Křížová L., Dadáková K., Kašparovská J., Kašparovský T. (2019). Isoflavones. Molecules.

[4] Zgórka G. (2009). Pressurized Liquid Extraction versus Other Extraction Techniques in Micropreparative Isolation of Pharmacologically Active Isoflavones from *Trifolium* L. Species. Talanta..

[5] Khazaei M., Pazhouhi M. (2019). Antiproliferative Effect of Trifolium Pratens L. Extract in Human Breast Cancer Cells. Nutr. Cancer.

[6] Booth N.L., Piersen C.E., Banuvar S., Geller S.E., Shulman L.P., Farnsworth N.R. (2006). Clinical Studies of Red Clover (Trifolium Pratense) Dietary Supplements in Menopause: A Literature Review. Menopause.

[7] Engelhardt P.F., Riedl C.R. (2008). Effects of One-Year Treatment with Isoflavone Extract from Red Clover on Prostate, Liver Function, Sexual Function, and Quality of Life in Men with Elevated PSA Levels and Negative Prostate Biopsy Findings. Urology.

[8] Kanadys W., Baranska A., Jedrych M., Religioni U., Janiszewska M. (2020). Effects of Red Clover (Trifolium Pratense) Isoflavones on the Lipid Profile of Perimenopausal and Postmenopausal Women—A Systematic Review and Meta-Analysis. Maturitas.

[9] Beck V., Rohr U., Jungbauer A. (2005). Phytoestrogens Derived from Red Clover: An Alternative to Estrogen Replacement Therapy?. J. Steroid Biochem. Mol. Biol..

[10] Occhiuto F., De Pasquale R., Guglielmo G., Palumbo D.R., Zangla G., Samperi S., Renzo A., Circosta C. (2007). Effects of Phytoestrogenic Isoflavones from Red Clover (*Trifolium Pratense* L.) on Experimental Osteoporosis. Phyther. Res..

[11] Baber R. (2010). Phytoestrogens and Post Reproductive Health. Maturitas.

[12] Rafii F. (2015). The Role of Colonic Bacteria in the Metabolism of the Natural Isoflavone Daidzin to Equol. Metabolites.

[13] Sun Y., Liu Z., Wang J. (2011). Ultrasound-Assisted Extraction of Five Isoflavones from Iris Tectorum Maxim. Sep. Purif. Technol..

[14] Pandit N.T., Patravale V.B. (2011). Design and Optimization of a Novel Method for Extraction of Genistein. Indian J. Pharm. Sci..

[15] Blicharski T., Oniszczuk A. (2017). Extraction Methods for the Isolation of Isoflavonoids from Plant Material. Open Chem..

[16] Rostagno M.A., Manchón N., Guillamón E., García-Lafuente A., Villares A., Martínez J.A. (2010). Methods and Techniques for the Analysis of Isoflavones in Foods.

[17] Nemitz M.C., Moraes R.C., Koester L.S., Bassani V.L., von Poser G.L., Teixeira H.F. (2015). Bioactive Soy Isoflavones: Extraction and Purification Procedures, Potential Dermal Use and Nanotechnology-Based Delivery Systems. Phytochem. Rev..

[18] Saravanabavan N., Salwe K.J., Sudar Codi R., Kumarappan M. (2020). Herbal Extraction Procedures: Need of the Hour. Int. J. Basic Clin. Pharmacol..

[19] Pham T.T., Shah N.P. (2009). Hydrolysis of Isoflavone Glycosides in Soy Milk by β-Galactosidase and β-Glucosidase. J. Food Biochem..

[20] Huang H., Liang H., Kwok K.-C. (2006). Effect of Thermal Processing on Genistein, Daidzein and Glycitein Content in Soymilk. J. Sci. Food Agric..

[21] Kar M., Chourasiya Y., Maheshwari R., Tekade R.K. (2018). Current Developments in Excipient Science: Implication of Quantitative Selection of Each Excipient in Product Development. Basic Fundamentals of Drug Delivery.

[22] Kerlin R.L., Li X. (2013). Pathology in Non-Clinical Drug Safety Assessment.

[23] Matulyte I., Marksa M., Ivanauskas L., Kalveniene Z., Lazauskas R., Bernatoniene J. (2019). GC-MS Analysis of the Composition of the Extracts and Essential Oil from Myristica Fragrans Seeds Using Magnesium Aluminometasilicate as Excipient. Molecules.

[24] Markl D., Zeitler J.A. (2017). A Review of Disintegration Mechanisms and Measurement Techniques. Pharm. Res..

[25] van der Merwe J., Steenekamp J., Steyn D., Hamman J. (2020). The Role of Functional Excipients in Solid Oral Dosage Forms to Overcome Poor Drug Dissolution and Bioavailability. Pharmaceutics.

[26] Jagtap P.S., Tagad R.R., Shendge R.S. (2019). A Brief Review on Kollidon. J. Drug Deliv. Ther..

[27] Kazlauskaite J.A., Ivanauskas L., Bernatoniene J. (2021). Cyclodextrin-Assisted Extraction Method as a Green Alternative to Increase the Isoflavone Yield from Trifolium Pratensis L. Extract. Pharmaceutics.

[28] Krähmer A., Gudi G., Weiher N., Gierus M., Schütze W., Schulz H. (2013). Characterization and Quantification of Secondary Metabolite Profiles in Leaves of Red and White Clover Species by NIR and ATR-IR Spectroscopy. Vib. Spectrosc..

[29] Sun J., Sun B., Han F., Yan S., Yang H., Akio K. (2011). Rapid HPLC Method for Determination of 12 Isoflavone Components in Soybean Seeds. Agric. Sci. China.

[30] Lante A., Barion G., Zannoni S., Rita M., Tinello F., Dal C., Mosca G. (2018). An Ecofriendly Procedure to Extract Iso Fl Avones from Soybean Seeds. J. Clean. Prod..

[31] Yoshiara L.Y., Madeira T.B., Delaroza F., Da Silva J.B., Ida E.I. (2012). Optimization of Soy Isoflavone Extraction with Different Solvents Using the Simplex-Centroid Mixture Design. Int. J. Food Sci. Nutr..

[32] Rostagno M.A., Palma M., Barroso C.G. (2003). Ultrasound-Assisted Extraction of Soy Isoflavones. J. Chromatogr. A..

[33] Ebringerová A., Hromádková Z. (2010). An Overview on the Application of Ultrasound in Extraction, Separation and Purification of Plant Polysaccharides. Cent. Eur. J. Chem..

[34] Vinatoru M., Mason T.J., Calinescu I. (2017). Ultrasonically Assisted Extraction (UAE) and Microwave Assisted Extraction (MAE) of Functional Compounds from Plant Materials. TrAC Trends Anal. Chem..

[35] Vinatoru M. (2001). An Overview of the Ultrasonically Assisted Extraction of Bioactive Principles from Herbs. Ultrason. Sonochem..

[36] Chiang W.D., Shih C.J., Chu Y.H. (2001). Optimization of Acid Hydrolysis Conditions for Total Isoflavones Analysis in Soybean Hypocotyls by Using RSM. Food Chem..

[37] Mathias K., Ismail B., Corvalan C.M., Hayes K.D. (2006). Heat and PH Effects on the Conjugated Forms of Genistin and Daidzin Isoflavones. J. Agric. Food Chem..

[38] Gikas E., Alesta A., Economou G., Karamanos A., Tsarbopoulos A. (2008). Determination of Isoflavones in the Aerial Part of Red Clover by HPLC-Diode Array Detection. J. Liq. Chromatogr. Relat. Technol..

[39] Fahmi R., Khodaiyan F. (2014). Effect of Ultrasound Assisted Extraction upon the Genistin and Daidzin Contents of Resultant Soymilk. J. Food Sci. Technol..

[40] Xu Z., Wu Q., Godber J.S. (2002). Stabilities of Daidzin, Glycitin, Genistin, and Generation of Derivatives during Heating. J. Agric. Food Chem..

[41] Vadlamudi M.K., Dhanaraj S. (2017). Significance of Excipients to Enhance the Bioavailability of Poorly Water-Soluble Drugs in Oral Solid Dosage Forms: A Review. IOP Conf. Ser. Mater. Sci. Eng..

[42] Vraníková B., Gajdziok J. (2013). Liquisolid Systems and Aspects Influencing Their Research and Development. Acta Pharm..

